# “*Your behavior is not welcome here*…”: forced internal displacement of sexual and gender minorities in Kenya

**DOI:** 10.3389/fsoc.2025.1499312

**Published:** 2025-02-28

**Authors:** Emmy Kageha Igonya, Ebenezer Kwesi Armah-Ansah, Winstoun Muga, Kristefer Stojanovski

**Affiliations:** ^1^African Population and Health Research Center (APHRC), Nairobi, Kenya; ^2^School of Public Health Sciences, University of Waterloo, Waterloo, ON, Canada; ^3^Department of Social, Behavioral and Population Sciences, Celia Scott Weatherhead School of Public Health and Tropical Medicine, Tulane University, New Orleans, LA, United States

**Keywords:** LGBTQ+, internal displacement, Kenya, stigma, sexual and gender minorities, migration

## Abstract

**Background:**

While migration studies have boomed, little is mentioned about internal displacement of queer persons. In Kenya, internal displacement of sexual and gender minorities is often overlooked and not well understood, which results in increased marginalization and vulnerabilities. The article provides an in-depth analysis of forced serial internal displacement trajectories of sexual and gender minorities, and its effect on navigating socialites and livelihoods.

**Methods:**

We draw on qualitative data conducted between 2010 and 2023 using participant observations, in-depth interviews, case histories, and focus group discussions with LGBTQ+ persons, as well as mothers and fathers of gay men in Kenya. We used a thematic approach and principles of interpretive anthropology to organize and describe the meaning of the data as a continuous process. Broader themes were identified from the beginning of the first study, resulting in the development of a codebook framework that was reiterated over time.

**Findings:**

Our findings show that sexual and gender minorities are forced into internal migration. Factors informing are located at the individual, interpersonal, institutional, and communal levels. We identified three main themes with eight subthemes. The main themes were (1) reasons for migration, which were often related to stigma, violence and oppression, (2) patterns of migration, which primarily had rural to urban patterns and instability, and (3) kinship, networks, and social systems, which required rebuilding, and gaining “respectability” from kin.

**Conclusion:**

There can be little doubt that sexual and gender minorities face stigma and discrimination across levels of the socioecological model, and that in most cases, there has been multiplied grievances and anti- LGBTQ+ activities instigating serial forced migration. Forced serial displacement of sexual and gender minorities in Kenya requires research attention, and it might be better served if examined through the lens of “forced migration,” given the non-voluntary aspects of fleeing and displacement. Rethinking LGBTQ+ IDPs through the lens of coercion may better capture the lived experiences given the structural stigma and violence in which they live and cannot escape.

## Introduction

This paper highlights a neglected area in queer migration studies in Africa. Internal displacement of persons is a global concern that warrants attention (Held, 2023; Danisi et al., 2021). More specifically, the forced serial displacement of sexual and gender minorities (SGM) or lesbians, gay, bisexual, trans gender nonconforming (LGBTQ+) people is a growing global phenomenon. Various factors, including stigma, discrimination, community surveillance, psychological harms from societal or family pressures and expectations, the need to balance between disclosure and being closeted, persecution, and violence, force LGBTQ+ people to flee within their own countries or across borders (Odlum, 2018; Hathaway and Pobjoy, 2012; Luibhéid, 2020; Shidlo and Ahola, 2013). These push factors highlight the suffering and structural violence inflicted on sexual and gender minorities. Other studies have highlighted emotional factors prompting LGBTQ+ migration, including seeking places where they can find other LGBTQ+ persons for a sense of belonging, freedom of expression, friendship, and psychosocial support (Stella et al., 2017; Gorman-Murray, 2009). While those fleeing seek freedom and security, cross-border migration studies also reveal they encounter challenges, such as violence, human rights violations, isolation, stigma, and discrimination, which aggravates and increases vulnerability and marginalization (Shidlo and Ahola, 2013; Ombagi, 2019; Zea et al., 2014). Other struggles include hostile situations, complex asylum and refugee-seeking procedures, access to basic needs, disruption of social networks, resettlement, integration, lack of income, loss of social support and poorer health outcomes, including mental health and safety risks (Camminga, 2018; Vogel, 2009; Logie et al., 2012; Piwowarczyk et al., 2017; Wijesiriwardena, 2022).

In Africa, anti-same-sex laws, combined with the portrayal of same-sex relations and gender non-conformity as Western ideologies that are un-African and anti-religious (Stella et al., 2017; Van Klinken, 2015; Tamale, 2017; Msibi, 2012), contribute to negative societal attitudes toward LGBTQ+ people. The religious and political class view LGBTQ+ as threatening Christian and African family values (Bongmba, 2015; Van Klinken, 2019). Among the general populace in Kenya, being LGBTQ+ evokes illegality, immorality, irreligiousness, and social deviance (Wilson et al., 2019; Republic of Laws of Kenya, 2010). However, LGBTQ+ people have gained visibility through HIV programming, which has become fruitful for activists mobilizing around human rights and social inclusion (National AIDS Control Council, 2009; NACC, 2009). The courts pronounced favorably on LGBTQ+ people’s freedom of association (Misedah-Robinson et al., 2022). Conversely, increased, visibility has sparked backlash and negative attitudes, (Misedah-Robinson et al., 2022; Mwaniki et al., 2024). Members of the LGBTQ+ community in Kenya regularly face stigma, discrimination, and human rights violations, including physical attacks, emotional and sexual abuse, arbitrary arrests, blackmail, ostracization, eviction from houses, dismissal from employment, and denial of justice (Wijesiriwardena, 2022; Lewis et al., 2023). This environment increases fear, vulnerabilities, and frustrations among LGBTQ+ people who flee hostile environments in search of security, freedom, and belonging (Msibi, 2012; Human Rights Watch, 2015; Moyer and Igonya, 2018).

Substantial LGBTQ+ migration research interest in East Africa has been directed to investigating cross-border, and mobility within urban spaces examining how LGBTQ+ people create and navigate LGBTQ+ spaces to understand their livability, which indicates potential opportunities and agentic power (Ombagi, 2019; Woensdregt, 2023). Research in Dar es Salaam, Tanzania identifies the possibilities of social support through kin-like relations in *‘bibi’* (older gay men) homes, providing support such as accommodation and food for new LGBTQ+ arrivals (Shio and Moyer, 2021). Woensdregt (Woensdregt, 2023) provides insights into how LGBTQ+ people in Kenya remake homes in urban areas and the desirability among LGBTQ+ populations to reconnect with their families. In a study with sexual minority asylum seekers and refugees in Nairobi, Kenya, the results indicated that continued and multi-layered discrimination across borders shapes adverse physical health outcomes for gay and bisexual asylum seekers and refugees (Wilson et al., 2019). The limited studies of LGBTQ+ migration in Africa often lack nuance or are missing from the migration literature and humanitarian response, particularly for internal displacement (Woensdregt, 2023; Marnell, 2023).

The socioecological model indicates that factors and interactions across the individual, interpersonal, community, institutional, and structural levels shape lived experiences (Kilanowski, 2017). We use the model to explore interrelated socioecological influences on the internal displacement of LGBTQ+ people in Kenya. At the individual level, LGBTQ+ individuals may grapple with internalized self-stigma and acceptance of their sexual and gender identities and secrecy around sexual orientation (Bhagat, 2018; Granderson et al., 2019). At the familial and communal level, queer people are confronted with extensive stigma often presenting in the form of sexual violence associated with traditional gender-based roles, physical assault, and discrimination (Lewis et al., 2023). At the societal level, the socio-political violence characterized by the criminalization of same-sex relations, anti-LGBTQ+ discourse among political and religious leaders, and misrepresentation of the LGBTQ+ community by the media also shapes exclusion and displacement (Bhagat, 2023). The lack of recognition of LGBTQ+ internal displacement exacerbates the complicated terrain of marginalization (Misedah-Robinson et al., 2022; Stojanovski et al., 2024)–(Quintero and Hari, 2022). In this ethnography of LGBTQ+ serial forced displacement, we aim to discuss the lived realities of LGBTQ+ people’s displacement by (1) identifying the drivers that make LGBTQ+ people flee in Kenya, (2) ascertaining patterns of movement during serial forced displacement, and (3) exploring how kinship, networks and social systems shape flights.

## Methodology

### Study design

We draw on five qualitative study designs, including longitudinal ethnography and cross-sectional studies conducted between 2010 and 2013. The topic of LGBTQ+ individuals and forced migration organically emerged and persisted in the five studies (or throughout these studies). It first appeared in 2010 during an ethnographic study of HIV care and social support for persons living with HIV in Nairobi (study one – an ethnography of HIV care study). In this study, MSM (men who have sex with men) sex workers were the primary participant group sampled and interviewed. The study site, known as Freedom Corner, was a designated space within a bar in Nairobi Central Business District, frequented predominantly by MSM sex workers. Freedom Corner also served as a meeting point for support group members, and, occasionally, visited by female sex workers and LGBTQ+ persons.

The second study, titled “HIV Key Population’s Bridging the Gaps,” was conducted in the Kenyan coastal city of Mombasa between October 2015 to August 2016 (Study two: Bridging the Gap study). Using a referent from study one, House K, located in a middle-income area of Mombasa and patronized mainly by middle and upper-social-class gay, bisexual, trans men and other MSM, became a regular meeting point and key study site. Discussions among participants frequently addressed forced serial displacement, including evictions, intra- and inter-town mobility, and migration from ‘*bara’* (mainland Kenya).

The third study, conducted between October 2016 and August 2018, was a two-year ethnography on the economic empowerment and political positioning of male and female sex workers (Study three: Economic Empowerment and Political Positioning of Sex workers). Of the 11 participants, five were MSM sex workers, four of whom had migrated from ‘bara’ (mainland Kenya).

The fourth study, “The Political Economy Analysis of Social Exclusion of LGBT’,” took place in 2020 across three Kenyan cities: Mombasa, Nairobi and Kisumu (Study Four: Political Economy Study). Discussions about serial forced migration continued to surface throughout this study.

The last study, “LGBTQ+ lived experiences study,” was conducted between 2022 and 2023 in Mombasa, Nairobi, Eldoret, and Kisumu (Study Five: Lived Experiences Study). It examined the lived experiences of LGBTQ+ people in Kenya.

### Study sites

Data were progressively collected in Nairobi (the capital), Mombasa a coastal city, Kisumu, and Eldoret (western Kenyan cities). These towns were selected because of the presence of LGBTQ+-led organizations and networks.

Nairobi County hosts the national government, county offices, most development partners, and civil society organizations (CSOs), including LGBTQ+ organizations. It is the most populous county, with an estimated 4.7 million residents (Kenya National Bureau of Statistics, 2013). It is characterized by diverse ethnic groups and livelihoods, with a significant portion of the population living below a dollar per day in informal, low-income settlements. The city has a sizable LGBTQ+ population and several queer spaces (Ombagi, 2019).

Mombasa, Kenya’s second largest and chief port, is a coastal county along the Indian Ocean. Its population is estimated at 1.3 million people (Kenya National Bureau of Statistics, 2013). Its economy is driven by tourism, trade, industrial manufacturing and fishing. In addition to the county government administration, it hosts some development partners and CSOs. It has a sizable LGBTQ+ population (Kenya National Bureau of Statistics, 2013) and male and female sex workers. Although it has a large presence of LGBTQ-led organizations targeting key populations, queer spaces for social gatherings are limited. The city has also seen a rise in anti-gay sentiment in recent years, as reported by local media.

Located in the Nyanza region near Lake Victoria, Kisumu County has a predominantly peri-urban population of 1.2 million (Kenya National Bureau of Statistics, 2013), with the Luo ethnic group as the majority. Fishing and sugarcane farming are the main economic activities. The region experiences high levels of poverty and food insecurity and reports the highest prevalence of HIV in Kenya, resulting in the presence of numerous of development partners and LGBTQ+-led organizations.

Eldoret, the headquarters of Uasin Gishu County, is in the Rift Valley region. The county has an estimated population of 1.2 million, with farming as its primary economic activity. Eldoret town, with an estimated population of 464,570, is cosmopolitan and peri urban. Although little is known about sexual and gender minorities in Eldoret, the town has a growing but discreet LGBTQ+ community. The site was recommended by LGBTQ+-led organizations in Mombasa and Nairobi.

### Sample population

The primary study population comprised LGBTQ+ individuals aged between 18 and 50. Participants in Study one included 24 men identifying as MSM and living with HIV (Igonya et al., 2022) who were members of a support group in Nairobi. Most were migrants from various parts of rural Kenya, while a few were born in urban areas. While MSM sex workers were a majority, the second, third, fourth, and fifth studies included LGBTQ+ individuals. Sex work was the primary source of income for MSM sex workers. The majority were members of LGBTQ-led organizations. LGBTQ+ individuals from the middle class had a higher education level compared to MSM sex workers and were employed either within or outside LGBTQ-led organizations. The sample also included five mothers of gay men who participated in a group discussion. Four of these mothers lived in low-resource settlements in Mombasa while the fifth resided in Watamu, a town 108 kilometers from Mombasa. All these women earned a living from petty businesses and casual labor. Two fathers from Mombasa also participated, one of whom was married.

All study participants provided consent to participate in the study. Study groups and participants were purposively and conveniently selected using snowball, LGBTQ-led organizations and network sampling (Coleman, 2017; Institute of Mathematical Statistics Stable, 2016).

### Data collection methods

The data collection techniques included participant observations, in-depth interviews, conversations, informal discussions, case histories, diaries, and focus group discussions. Participant observations conducted between 2010 and 2023 were the primary data collection technique. These were used during deep-hanging sessions with participants in various spaces and study sites, including regular meeting venues, advocacy activities, and funerals of LGBTQ+ people. Accompanying participants’ observations were conversations and informal discussions intentionally orchestrated for clarifications and to prompt in-depth discussions. Field notes from participant observations and conversations were jotted down and elaborated on at the end of each day (Emerson and Frits, 2005). Cumulatively, the study’s data corpus draws from 163 participant observations accompanied by conversations and informal discussions conducted mainly at Freedom Corner, House K in Mombasa, and at the LGBTQ+ organizations in Eldoret and Kisumu.

All 24 Freedom Corner members (HIV-positive MSM sex workers) participated in in-depth interviews in study one—ethnography of HIV care. Data was collected through participants’ observations, in-depth interviews, two focus group discussions, and case histories. Conversations and informal discussions were also included.

For study two—bridging the gap study, data was collected through four case histories, participant observations, 14 in-depth interviews and two focus group discussions. In addition, a group discussion was conducted with mothers of gay children in Mombasa during the second study.

In study three—the economic empowerment study, five economic diaries were the primary data collection tool during the study on economic empowerment and political positioning of sex workers’ study. The diary biweekly meetings were accompanied by informal discussions that built up case histories. Often, conversations on forced migration popped up in these discussions, mainly focused on participants’ families. Those who took part in economic diaries were included in life histories. Ten informal discussions were conducted with MSM from diverse socio-economic status.

In study four (the political economy study) and study five (LGBTQ+ lived experiences), data was collected through FGDs, in-depth interviews, key informants and case histories. Key informants included community leaders, religious leaders, civil society organizations, and representatives of county and national government sectors. These interviews were conducted virtually.

In study four and five, a total of 108 in-depth interviews were conducted with the following breakdowns: Nairobi (*n* = 47), Mombasa (*n* = 27), Kisumu (*n* = 24), and Eldoret (*n* = 10). In total 34 focus group discussions were conducted in Nairobi (10), Mombasa (8), Kisumu (8), and Eldoret (6). Sixteen case studies were conducted in Nairobi (12) and Mombasa (4) during the first and second studies, respectively. Focus group discussions and in-depth interviews were conducted in all studies. These were a one-off encounter, while life histories involved a series of back-and-forth interviews and conversations on mobility, social networks, and connecting with kin. In-depth interviews and discussions increased during the studies on political economy analysis and LGBTQ+ lived experiences. Throughout the studies, recurring themes included forced migration and its implications for LGBTQ+ individuals (Table 1).

**Table 1:** Summary of studies, sites, participants and data collection methods.

Study	Year of data collection	Site	Participants	Methods	Study design	Research team
Study 1: The Emergence and Implications of Support Groups in the Care and Support for People Living with HIV in Kenya.	2010–2014	Nairobi	MSM living with HIV	Participant observations IDIs case history FGDs	Ethnography	First author
Study 2: HIV Key population in Bridging the Gaps operational study	2016/2018	Mombasa	MSM, and sex workers Mothers of gay children Fathers of g	Focus groups discussion with MSM Focus group with mothers of gay children. IDIs participants, IDIs with two fathers of gay children.	Cross sectional qualitative	First author and a research assistant
Study 3: Economic Empowerment and political positioning of LGBTQ	2016/2018	Mombasa Nairobi	MSM sex workers, -follow on with Freedom Corner group from 2010	Participant observations Case history/ economic diaries, Informal discussions	Ethnography	First author
Study 4: Political economy analysis of social exclusion of LGBTQ	2020	Mombasa, Nairobi, Kisumu	LGBTQ+	Community-mapping FGD	Cross sectional mixed methods	Three researchers Three research assistants
Study 5: Lived experiences of LGBTQ individuals in Kenya.	2021/23	Nairobi, Mombasa, Kisumu Eldoret	LGBTQ+	IDIs FGDs Life histories	Cross sectional mixed methods	Three researchers 8 research assistants

### Data analysis

Data analysis was a continuous activity throughout the life of the research projects (Mills, 2019). We used thematic approach and principles of interpretive anthropology to organize and make meaning of the data (Geertz, 1983; Osorio-Parraguez, 2013). Broader themes were generated from the beginning of the first study, resulting in the development of a codebook framework capturing and defining themes and subthemes, including identifying patterns and recurrent issues (Braun and Clarke, 2019). At the end of study 2 and 3, data were transcribed and inputted into NVivo software. The transcriber and first author familiarized themselves with the data and iteratively reflected on and expanded themes from new data. At the end of study five, two researchers and two coders read through two randomly selected FGD, IDI transcripts and fieldnotes from all the studies, and using the developed coding framework inductively identified and refined labeling, (re)defined themes and sub-themes and patterns, and expanded the framework. Attention was paid to ex-post analysis involving importing and coding all data NVivo 12, a computer-assisted qualitative data analysis software (Dhakal, 2022). Using the coding framework, all data were coded in NVivo 12.

## Results

We identified three main themes with eight subthemes. The main themes were (1) reasons for migration, (2) patterns of migration, and (3) kinship, networks, and social systems. The subthemes included (1) community surveillance, stigma, and displacement; (2) patriarchy and misogyny; (3) disclosure and secrecy; (4) urban mobility and (in) stability; (5) Making and navigating internal displacement movements; (6) economic and social survival; (7) (re)building nodes and networks, and (8) respectability and (re)connecting with kin. These themes, illustrated through excerpts from the data, function together to provide insight into the complex LGBTQ+ internal displacement.

### Reasons for migration

Factors that shaped LGBTQ+ migration were numerous, but we focus on three main subthemes: (1) community surveillance, stigma, and displacement, (2) familial and sociocultural expectations, and (3) disclosure and secrecy shape internal displacement of LGBTQ+ individuals.

### Community surveillance, stigma, and displacement

Most forced migration was driven by perceived and enacted threats and violence, as well as stigma and discrimination from their families and community members. Two days after the death of a well-known LGBTQ+ activist in 2022, Mark (he/him), from the same geographic location, was fleeing from town A to town B after receiving threats from community members. Still, town B was not very safe, and Mark had to move further to town C. Mark lamented the hostile political rhetoric and rising tensions in the general community toward the LGBTQ+ community in town A. Receiving life threats forced Mark to migrate for safety. He stated, “People are talking bad about sexual and gender minorities and me. It is all on television, everywhere. The environment is hostile for now. I left, and I’m in a safe house in [City B].” Two months later, the first author called to check on Mark. He moved to Town C. *Fieldnotes, 2022- study 5.* While many leave, not all do so. A spot check in town A, 3 months later, a university student who identified as gay sobbed when narrating how he was verbally abused by strangers while he was walking, which made him very scared for his life. “I was passing, and a group of people started shouting, see this one who walks like a woman, he is one of them… I was very scared and walked past them hurriedly without saying a word.,” *Fieldnotes, Nairobi, 2023- study 5.* The harassment and surveillance stories of displacement were not unique.

In November 2010, the first author met Mary, a gay man, who introduced himself by a female name (here forth Mary) on the second visit to Freedom Corner 2010. Mary (she/her) was quiet and calm, holding a cell phone. After exchanging pleasantries and Mary introduced herself. ‘I’m Mary from Western Kenya’. She went on to narrate her journey of displacement from a rural area to Freedom Corner in Nairobi,

“I left my village after some people told me they would beat me up if I did not leave the village. I was scared. When I was in standard seven in primary school, I made a sexual advance toward certain boy I liked very much, and he reported me to those villagers. … One day, on my way from school to home, four men stopped me and told me I would regret it if I did not leave the village. One of them said, ‘Your behavior is not welcome in this village.” … We will come for you if you do not leave.’ One of them chased after me … and I ran faster than him …. I was fearful. I did not know what would happen to me next. I did not go home. I was afraid that they would report me to my parents, and I did not know how my parents would react. … Instead, I ran to my maternal grandmother’s home, about three kilometers away … I knew I could not hide longer …. I was to run off to a faraway place where I could not be traced.” – *IDI Nairobi November 2010 -study 1*.

Many of the Freedom Corner members, those visiting Freedom Corner space, and those with whom the first author subsequently interacted with in other cities recounted similar experiences. They ran from their rural homes to the city and towns after receiving threats from community members. Meggie (she/her), also a gay identifying man who presented with a female name, dropped out of school at 16 years and ran away from her rural home in Meru to Nairobi. She fled the countryside after a group of men in the village harassed her. The man told me, “Behave like a man, not a woman, or else we will teach you a lesson.” *Fieldnotes, Nairobi November 2010- Study 1.* Similar stories were shared in Mombasa, a coastal city in eastern Kenya, where participants discussed their flights in focus group discussions and in-depth interviews. James shared that he had flown from a village in rural Kisumu in Western Kenya after threats from villagers. “I was walking around the village when a group of men approached me and told me to either leave the village or they will burn me alive.” - *Fieldnotes, November 2010- Study 1.*

Some of the LGBTQ+ people ran away from forced conversion therapy to free them from demons, or were excommunicated. Two years after my encounter with Freedom Corner, Catherine (she/her), who was usually cross-dressed in tight feminine jeans, a jacket, and a handbag, disappeared. A year after her disappearance, she showed up during a sex work advocacy activity at the original Freedom Corner in Nairobi’s Uhuru Park. Catherine had changed her way of dressing. She was in an oversized long-sleeved shirt and a buggy pair of trousers. I asked her what happened to her. She explained how she went underground after her pastor asked her to leave the church:

“The pastor came in during choir practice and said, ‘a gay person in this choir should leave.’ No one responded. He repeated, ‘a gay person in this choir should leave.’ Again, no-one responded. On the third time, I walked away. I left church. I was so hurt and felt so ashamed that my secret was out. I moved to another part of town and stayed low key.” *Fieldnotes, Nairobi, 2023-Study 1.*

Those in church choirs expressed being ordered to leave the church while those attending church concealed their sexual and gender identities. Verbal and physical threats from fellow churchgoers were a familiar story told related to fleeing and displacement. Some reported death threats if they did not leave, while others were [literally] chased away. The lives of those forced into migration changed on the spur of the moment, a characteristic of all those whose displacement were sparked by neighbors, family, and the larger community.

### Patriarchy and misogyny

Another subtheme for reasons for displacement were familial and social expectations, which play key roles in queer flight trajectories. Social and gender norms and expectations were often at the root of the family “conflict” with LGBTQ+ family members.

Lucy (she/her), from Western Kenya and living in Mombasa, was kicked out of her home by her siblings after the death of her mother. She shared,

“Soon after the burial of my mother, life changed drastically. The people I thought were my siblings disowned me. I was the last child in the family. My father died when I was very young. The day after the burial of his mother, I was chased away by my siblings, who claimed that we could not be from the same father because I’m a boy who behaves like a girl. I wondered what had happened because I was their brother before our mother died, and now they disown me. I clearly remember that morning. My elder sister gave me my clothes and told to leave the house; they did not want me around. It was sad. I did not expect this, and none of my siblings stood up for me.” *IDI, Mombasa, 2016-study 3.*

In Nairobi, at the Freedom Corner, Mary, who lived in a makeshift park, shared how she had taken in a middle-income gay man after he was disowned and chased away by her family members. ‘The young man is in college, and he was told by his parents and siblings that he should stop such behavior [being gay] then they can support him.’

On February 20, 2017, in a focus group discussion, gay participants in Mombasa sobbed while intensely discussing how their fathers chased them away with or under the watch of their mothers, whom they believed loved them, “After my father discovered I was gay, he chased me away. I thought my loving mother would talk to my father about such a decision. She did not. Instead, she echoed her husband’s decision,” -*FGD participant -study 2*. While mothers were not overtly hostile, participants intimated that mothers did chase their children away via other forms of ostracization and gender role demands. “As for me, my mother has not chased me away, but the time I visit, she comes up with stories about marriage, saying that my agemates are now married or have children. I would walk away. But now I do not go home anymore. I am planning to have an arrangement to have a child with a female sex worker. Then I can go back home. That is how mothers send us away,” noted Kathleen (she/her) -*Fieldnotes- 2016. -study 2*. One of the participants who remained in contact with the mother by phone noted how the pressure by the mother to bring home a [heterosexual] wife has had negative implications on their communication, “I communicate with my mother, I miss her, but she always tells me to bring her a girl [female wife].” -*FGD Mombasa -Study 2.*

On the other hand, gender norms and patriarchy created conflict within families, and often, mothers were “to blame” for their child being gay. Such mothers took flights with their children. On March 17, 2017, the first author met with the father of a gay son for a scheduled in-depth interview. After the introduction of the study and consenting to participate, an angry-looking man explains how his wife left with [their] gay child. Fuming with anger and in a loud voice blaming the wife, he explained:

“I met my wife here in Mombasa. We did not know each other. I did not know her family. We moved in together and started a family. We both hustled selling second-hand clothes. While hawking, some men commented that you do not know your son well. I did not understand. One day I came back home and found a man on top of my son. I was very angry. I beat my son thoroughly, and I think I broke his wrist. My wife […] was very angry that I beat my son. She left with the son. You see, she knew the son was *shoga* [gay] all along. They left town and blocked me. I do not know where they are. But I can tell you, it is not in my generation. I have researched, and we do not have such behavior. It should be in my wife’s generation, and that is why she left.” *IDI, Mombasa, 2016-study 2.*

Similar stories arose from other participants. Some mothers lost their marriages when fathers of their children chased away queer children away, insisting that the mothers were the source of their son’s gay behavior. The first author first heard about a mother in flight with a gay child in Nairobi in December 2017. Joy, gay (he/him) shared how his father chased away his mother with her children:

“I really love my mother. She loves me too. When I was 8 years old, and started to show girlish traits, my father chased away my mother with all her children, saying, ‘I did not marry you to give me such children.’ That is how my mother left the village, and we moved to Nairobi.” *IDI, Nairobi, 2017 -study 2.*

In an FGD in Mombasa, mothers of gay sons shared how they were put out of their homes by their husbands for giving birth to gay children. Jane sobbed as she explained how she, together with her children, were furiously put out of their house by her ex-husband:

“We [participant and former husband] were pastors. When we got married, we took some time to conceive. We prayed, and we were blessed with a baby boy, and then, after a year, we had a girl. My husband came home and found our son in bed with another man. He telephoned [me] and told me to come home immediately. When I arrived home, he said, ‘A child of Satan is Satan. I do not want to have anything to do with you and your children.’ He was outraged and did not want to listen [to reason]. I took the children and ran to my in-laws. However, I found they were told not to allow me in [sobs].” *Informal discussion, Mombasa, 2017 (study 2).*

Similarly, Fiona, a mother of a gay child, narrated how she is tired of mobility because of their gay son. “I have moved from one area to another in this [Mombasa] town. I’m tired. My husband chased me away with all four of my children. When I told him it was only one child with the problem [gay], he told me, ‘All your generation is contaminated. Take all your children with you.’ I left with the four children, and I have been moving from place to place for the security of my son because when neighbors get to know my son is gay, they tell me to take my son away from them because he will *‘spoil’* their children. Some of the neighbors have threatened to beat him.” - *Informal discussion, Mombasa, 2017 study 2.*

The experiences of Betty, Fiona, and Joy’s mothers and the women in the FGD demonstrate how society continue to blame and punish mothers for children’s “defects.”

### Disclosure and secrecy

Disclosure and secrecy were other push factors for the displacement of queer individuals. Deciding whether to disclose or not was frightful, fueled by due to the uncertainty and associated risks. As explained by a participant in Kisumu:

“It is a tough one. In my case, it was worse because when I came out a few years ago there was no exposure [to topics of LGBTQ+]. They expect you to be a man. I told them … I love men, yet you are a man! It hit them so hard, and it led to me being excommunicated by the whole family; I was discriminated against. So, for like 10 years, I have not been close to my family, yeah.” -*In-depth interview, Kisumu 2020- study 4.*

In Nairobi in September 2016, Jane, a lesbian working with an LGBTQ+-led organization, shared how her father told her to leave his home in Murang’á in Central Kenya because she was a shame to the family. “You know I had not told my father about my sexuality, and then on this day, he just confronted me saying, ‘What is this I have heard about you?’ I asked him to tell me what he had heard about me. In response, he told me, ‘I want you to leave this home; you have shamed the family.’ That was it. I had some money, and on the next day I left for Nairobi, rented a place, and here I was.” *Fieldnotes, Nairobi, 2016 -Study 1.*

Participants contemplated the pain of rejection. Some adopted secrecy as a survival strategy within families, doing everything possible to conceal their sexual and gender identities. However, secrecy was not easy to maintain, it was painful and draining, which would shape the desire and push toward flight. FGD participant in Nairobi (Study 5) explained, “When you are not free within your family, you isolate yourself, and you become too over-conscious and irritable. You are there but not with them. It is a burden to yourself. You tell many lies, many excuses, in the end you either walk away or you hear someone committed suicide.” Some participants were taken for “healing” prayers before they ran away. In July 2021, a Pentecostal pastor in Kisumu narrated how the son of a prominent political individual came out to the family, and this pastor was called to pray for the child. The son told the pastor, “This is how I feel. I’m gay, and if my father cannot accept me, will other people out there accept me?”- *Key informant- study 4*. In this case, the son did not run away; instead, unfortunately, a month later, the pastor was informed by the politician that his son committed suicide. This experience conveys a sense of need for support to facilatate the disclosure process for both LGBTQ+ and their family members.

In June 2022, the first author on research fieldwork supervision met Sydney, who hails from Vihiga Country and is a peer educator at the LGBTQ+ organization in Eldoret. Sydney explained how his flight was influenced by secrecy around his sexual orientation:

“At home, we always watched the news on television as a family. Especially the evening news. Now, this time, there are LGBTQ+ people, and from my parent’s comments, I realized how they hate LGBTQ+ individuals. ‘These are confused people who are learning foreign behaviors. They should be killed’, said my mother. I did not talk. I was very depressed. I do not know what their reactions would be if I came out to them.” *Fieldnotes, Nairobi, 2022-study.*

Concerns like these [talking bad, negative attitudes toward LGBTQ+] are common, and a clear contribution to secrecy and internal displacement of LGBTQ+ individuals Sydney’s experience demonstrates that fear of the consequence of (un)intended disclosure drives flight in pursuit of secrecy and safety. Many participants alluded to the secrecy strategy to avoid discrimination and loss of housing, education, and access to family support. This meant concealing anything that would lead to disclosure, intended or unintended.

While disclosure is frightful, from the political economy study [study 4] with LGBTQ+ persons in 2020, some had good experiences with disclosure. Some levels of acceptance or tolerance in families that have happened in the last 5 years were shared:

… expressing my sexual orientation and identity has not been a challenge so much because I had cooperative parents. My family understands who I am, and they embrace my queerness, and access to education has not been such a challenge because I had the privilege to go to school, I had the privilege of [fees] being paid for, I had the opportunity to go to college, and I had the opportunity to work. *-PEA in-depth interview, Kisumu, 2021-study 4.*

Participants attributed such changes to advocacy and training milestones in community awareness and service delivery that have reduced stigmatization and discrimination. Participants were hopeful that society is becoming more knowledgeable and understanding of sexual orientation and gender identities:

… I have received support from family and friends: I have received major support from friends, well-wishers and organizations. *In-depth interview, Nairobi*- *study 5.*

Nevertheless, this is not the norm. The same political economy (study four) and LGBTQ+ lived experiences (study five) studies show pervasive negative attitudes toward LGBTQ+ people prevail in families, society, livelihoods, and service delivery, leading to greater secrecy. “It is not a smooth line. [Be]cause, sometimes when you get a job, you must hide because if they find out about your sexuality, sometimes they tend to pull away from giving you some responsibilities.” *In-depth Interview, Mombasa-study 4.* The dilemmas around coming out [disclosure] are a characteristic of a problematic affair.

“… I mean, if you are an LGBTQ+ person and do not disclose yourself, then things may work out for you. It will not be easy when you expose yourself.” *In-depth interview, Kisumu, 2022-study 5.*

To disclose or not remains a daunting task to many: the decision to disclose is complex, especially where disclosure would be used against those who disclose. Discussions in the focus groups and in depth interviews emphasized dilemma with the outcomes of disclosure:

“Worse, it is a tough one. But like, in my case, it was worse because when I came out a few years ago, back then, there was no exposure. They expect you to be a man. I told them … I love men, yet you are a man! It hit them so hard, and it led to me being excommunicated by the whole family; I was discriminated against. So, for like 10 years I have not been close to my family, yeah.” *In depth interview, Mombasa, 2022 -Study 5.*

## Patterns of migration

### Urban mobility and (in) stability

The flow of LGBTQ+ people from different parts of the country to towns or cities may make urban areas seem safer. Narratives of LGBTQ+ inter-urban mobility and, intra-city migration was common.

To understand the grounded urban (i)mobility requires the individual and collective of the social and economics that run through towns. When the first author arrived in Mombasa for study 3, Musa (he/his/him) invited her to his spacious two-bedroom apartment located in an up-and-coming modern apartment building. Musa, originally from Western Kenya, moved from rural to urban areas after his father chased him away following his expulsion from school for unbecoming [sexual] behaviors. Musa was a week old in this apartment. He moved here after an eviction by the landlord. He blamed the movements on his sexual orientation. He narrated in an informal discussion how he received a letter from the landlord asking him to vacate the house. “I’m a week old in this apartment. I was evicted by neighbors [at my previous one]. The landlord sent me an eviction letter.” While the letter was from the landlord, Musa intimated that his neighbors [fellow former tenants] must have put pressure on landlord to evict him because of his perceived queer identity. Gay, bisexual men, and transgender people who participated in an FGD in Mombasa in January 2017 discussed surveillance in low-income Swahili houses and in middle- and high-income areas forcing them to flee.

The Swahili house structures are rectangular shaped, with one main entrance door leading to a rectangular free space in the middle and a shared kitchen at one end. The house has several single rooms with doors facing the rectangular free space. Those renting rooms usually sit outside their doors occupying the free rectangular space to chat and gossip. This space provides a window for surveillance. Neighbors watch each other’s movements in and out of rooms including visitors. According to the group discussion with men who have sex with men in Mombasa, neighbors confront those with unbecoming behavior, including their sources of income, marriage, and visitors. “They watch everything … everything, and they keep track including the time you are in your house, when you go out and when you come back, who is visiting your house (room), and they ask questions like why you [a man] cook and wash clothes when there are so many women, or when is your girlfriend visiting.” – *Informal discussion Mombasa -study 3*. This is a daily experience for LGBTQ+ IDPs. Some of LGBTQ+ individuals opt to move out when the surveillance is too much, this was perceived as the best option in terms of safety and opportunities.

Phased or multiple flights were common. Lucy, like Karen and Musa, has experienced multiple displacements. She had moved six times since she arrived in Mombasa from rural western Kenya. During the second study, the first author visited the spacious self-contained room Lucy rented in Mombasa. Her room was well furnished with a five-seater sofa set, a television, a gas cooker, and other basics. She had just been chased away by irate neighbors when the first author was back for the third study. Lucy explained:

“Two weeks ago, a group of my neighbors came to my house and told me to leave the neighborhood because *mimi ni shoga* (I’m gay), therefore a bad example to young people and children. I realized my life was in danger. I locked the house and left. I did not take anything. I did not argue with them. I walked away as they hurled insults at me, some saying that I should never come back, or they did not want to see me around. I boarded *a matatu* (public transport) to town to process what had just happened.” *-Fieldnotes Mombasa 2017-Fieldnotes Mombasa- Study 3.*

Together with the researcher, Lucy, in trying to access her house to collect her personal belongings, could only watch her house from a kilometer away. Participants in all study sites made reference such eviction and community policing. In Nairobi, most Freedom Corner group members alluded to multiple movements within Nairobi city in pursuit of safety. Narratives of those who lived and moved in and out of Eastlands of Nairobi were dominated by the Mungiki, an outlawed ethnic gang in Kenya. While LGBTQ+ living in low-resource settings in Nairobi accounted for many of the displacements, some of those living in middle- and upper-income areas were not spared. During the political economy and LGBTQ+ lived experiences (study 4 and 5), FGD and in-depth interview participants in all study sites lamented a great many evictions. Their major concern was security. On February 2, 2024, the first author received a distressing call followed by a text from Khadija, one of the Freedom Corner members. Khadija (participant in study 1,4 and 5) was admitted to Mbagathi Hospital in Nairobi with injuries on his genitalia inflicted by a motorbike “boda boda” Uber rider. Khadija explained, “After drinking in one of the LGBTQ+ joints in Nairobi’s suburb at around 10 pm, I ordered an Uber ride to take me home. The Uber rider turned assailant took a different route and chopped my penis, saying, ‘Your people [LGBTQ+] are increasing in numbers in this place, we do not want to see you here.” Khadija’s situation highlights how unregulated community surveillance on LGBTQ+ is infiltrating societal structures. Participants reflected on evictions and violence as a sense of deficiency in laws and community support.

The challenges that trans people were experiencing were unique and greater than those encountered by the lesbians, gay and bisexuals. A group of trans women narrated in a FGD in Mombasa:

“R1: Yeah. Housing is a challenge because when people are looking for houses, and you approach a landlord, and they look at the way you appear, they set rules that do not exist. It is a challenge because where people live, most LGBT people do not stay in their houses for more than 3 months. Someone will stay in a house for a month, and then the neighbors will start complaining to the landlord.”

“R3: To add to what my friend said, there is a lot of discrimination. Let me talk on behalf of trans women. There is a lot of eviction. When you start transitioning, my life before was maybe a man or a gay person. But then you begin hormonal therapy and start developing breasts, and the complexion changes. You have done the social transitioning, and now you do the health transitioning, so you get people who do not understand you. So, you are evicted, house to house, and the eviction is monthly.”- *FGD Mombasa -study 5.*

Contributing to narratives on frequent evictions and mobility, the FGD shows how appearances of trans, particularly those who have or are transitioning are a determinant in eviction and violence.

LGBTQ+ individuals forced into perpetual mobility cycles, moving from one part of the country to another, or from different parts within the same city is a clear indication of the conflict between society and institutions on one hand and, on the other hand, sexual and gender minorities.

### Making and navigating internal displacement movements

Most flights were instant, and abruptly executed. The nature of threats, urgency, and the execution of evictions, elevated the likelihood of instant flights. Economic opportunities were not a strong determinant for first-time moves, especially if it is life threatening. Often, LGBTQ+ people moved in phases due to limited resources. In the first phase, they moved to nearby places where they could get work, save money, and then eventually move to desired destinations. Lucy, for example, had nowhere to go when she was chased away from home. She did not have money for her movement. While she wanted to be far away from her family for confidentiality reasons, her choice to move to Mombasa was influenced by people in her community who previously lived in or visited this destination. She walked to Kisumu, 10 kilometers away from her home. She secured casual jobs, mainly carrying goods for traders in the market to raise money. Two months later, she saved enough money for her flight to Mombasa. Similarly, Mary and others at the Freedom Corner and various study sites discussed how they moved to a nearby location and took up casual jobs to raise funds to facilitate movements to desired destinations. Eviction means leaving all your belongings behind and starting life afresh elsewhere. You are not allowed to collect your belongings. It is running away for your survival, said Lucy in Mombasa. When evicted from their houses, some end up sharing cheap hotel rooms with friends, others are accommodated by friends for a few days while they accumulate money to rent own houses within the same towns or preferred destinations.

However, few planned to exit their families and communities. Often, they used networks, including friends, to support their flights. The networks were critical in organizing for forced migration and fleeing, including planning the timing financial support and decision making on choice of destinations and accomodation. A case in point is Sydney who was influenced by information from friends on places that could enhance secrecy. Jalida, a lesbian, who lived in Nairobi, explained how her parents chased her away and how a friend helped in planning her flight and timing. “I was very confused. I was not prepared. I called a friend, a former beneficiary of J’s house, who directed me to J’s house.” Jalida slept at a friend’s house, and since she had money, she left for Kisumu County the following day. J’s house provides shelter, food, and psychosocial support to lesbians in flight.

The choice of destination for most of the younger LGBTQ+ in flight was made by their mothers, who were concerned with the safety of their children and avoiding stigma. Most mothers moved to unfamiliar areas of towns or cities that promised safety and confidentiality. Within cities, mothers were on perpetual move. As discussed above, after being chased away by her husband for giving birth to a gay child, Fiona reported multiple flights within Mombasa town. Their mobility is prompted by fear of possibilities of [unintended] disclosure. Fiona, like the mothers, moves when being undercover becomes impossible. While most mothers concentrated on their movement within cities, some moved to other towns. Joyce, the pastor’s ex-wife, together with her two children, became homeless after her husband threw them out, and with the financial support of a friend, she moved to another city:

“That day I slept on the streets. [sobs for seven minutes] I called a friend who gave me some money. I moved to Watamu [a coastal town]. I called my sister, who gave me some money to pay for a hotel. The next day, I got a cheap room and paid Ksh2000 (~$16) for a month. I also found some housework for Ksh500m (~$4) per day. I got a school for children. Three years later, I got a boyfriend. I got a house for my son about three kilometers away, and he moved out. I did not want my boyfriend to meet my son. I was afraid to tell him about my son in case he found out he was gay and left. When he [son] completed school, I took him to Malindi town, where he lives. He is now working.”

The choice of destinations for other younger, isolated gay men fleeing was influenced by their social support, mainly their older boyfriends, who offered them free accommodation and food. Sixteen-year-old Juma, born and raised in Mombasa, was in high school when he was forced to flee to escape his father’s rage and threats to kill him. At the time of the interview, Juma was living at the boyfriend’s house. Juma explained how, while walking from school to home, he met his mother along the way, telling him to run away. “She told me, ‘My son, I love you. Run away from your father. He is very outraged with your gay behavior and has sworn to kill you.’” Juma ran to the boyfriend’s house in Mombasa in an area he thought would hide him from his father. Discussions around young queer persons in displacement alluded to Juma’s experience. Discussants indicated that cases of younger queer in flight were few, and most of the younger one was taken in by older queer boyfriends or friends, particularly those who their mothers did not accompany.

Intra-town or city-to-city migration is the least common and usually marked by temporality. Unlike rural–urban migration, urban-to-urban movements were for a shorter period. Choices were made based on economic ability or familiarity of friends or social networks and queer organizations. At Freedom Corner, it was common for LGBTQ+ people from other towns to come through; some were visiting for a few days, while few were relocating. Others moved back and forth between cities mainly in pursuit of safety. When Kate won the 2016 Mombasa Mr. Red Ribbon competition, a photo of him receiving the award went viral on social media, sparking a community threat toward him. Mr. Red Ribbon is a yearly event aimed at reducing stigma and discrimination among people living with HIV, whereby, through competition, MSM in various towns elect their HIV ambassador. Following Kate’s winning, a group of enraged neighbors came to her house, demanding that she vacate the premises. She fled and relocated to the neighboring town of Malindi before making her next move. Three months later, she relocated back to Mombasa, but to a different part of Mombasa town.

While safety was the foremost, financial ability was also crucial in choosing destinations. Some of the organizations have finances and safe places earmarked for sexual and gender minorities facing violence. Mark’s flee trajectory is a case in point. When Mark came to town B, he lived in a safe house, and when town B became unsafe, he quickly moved to town C. Such support is only for a few days. However, according to many study participants, the safe houses only benefit activists or staff of LGBTQ-led organizations, and only a few are evacuated by LGBTQ-led organizations. Lucy, in Mombasa, lamented how for 2 weeks, she tried to get the support of queer organizations in vain:

“It has been 2 weeks since I was chased away from my house. I approached organizations here in Mombasa to help me move to a safe place or even move my personal effects in vain. I sought help from these [queer] organizations, but they did not help. Yet when Musa was kicked out of his house, queer-led organizations supported him. They got him a home, paid and moved his personal stuff. This is because he is one of those leaders in these organizations. But for me, I have tried to get help from them in vain.”

Such sentiments are shared among queer individuals who are not activists who feel they do not get the help they need when experiencing violence, including evacuation, safe spaces, or legal representation. Additionally, each town had a distinctive relational culture. For instance, Nairobi was somewhat of a socialist culture, where it was common to request financial support and share rooms with friends or through social networks. Mombasa, on the other hand, was more capitalist; people typically lived in their own houses or rooms, paid their bills independently, and occasionally relied on loans when seeking financial assistance. Such cultural differences are limited urban to urban mobility.

### Economic and social survival

For most displaced LGBTQ+ Kenyans, economic precariousness was a concern.

“… it becomes a … challenge in terms of earning a decent living. Or just a livelihood that can enable you to buy clothing, buy food, or even pay rent. We are struggling with work. It will be tough, but there is progress*.,” In-depth Interview, Kisumu, 2022*.

Constant mobility exacerbates economic hardship and lack of social support. “When you leave in a hurry without anything, no money, no place to stay, but you just leave for unknown, nowhere to report or seek help from, yet maybe you have no education certificate,” lamented an in-depth interviewee in Nairobi. Most of those fleeing faced social misery. Livelihood and social navigation in flights were widely discussed at all study sites. In Mombasa, however, there were discussions on businesses and jobs in the hospitality industry given the coastal tourism economy, even though they did not escape sex work and depended on the limited NGO- economy.

For most of the study participants, sex work is a core aspect of LGBTQ+ serial forced displacement survival. In Nairobi, sex work was often discussed or negotiated at the Freedom Corner. This was also common in Kisumu and Mombasa. Most participated in sex work as a primary source of income, which has remained so for many participants. “My peers mostly meet their livelihood through sex work, and it is sustaining for them mostly,” a participant in Mombasa stated. Income from sex work catered for basic needs for the majority. “Most of them engage in sexual activities to earn money or maybe to get food, get a house, and all that,” a participant from Kisumu explained. From conversations, sex work was an immediate survival tactic, not really a free choice but coerced, given the need for survival. As illustrated by Mary’s experience from western Kenya, who arrived in Nairobi late in the evening and had nowhere to go:

“It was my first time [in Nairobi]. I knew nowhere. I was a stranger. I walked around town. It was very confusing. Many people and vehicles. I stood outside some shop for some time, and a security guard asked me what I was up to. I explained that I arrived from a rural area, and I was lost in the city. He offered me a place to sit. As he left in the morning, I also moved around town and ended up at the ring-shaped place outside Hotel X. Many people, the majority of whom were men and a few women, sat on that ring idling. Evening came, and a man in a car beckoned me to go to the car [laughs]. He asked me what I was doing at the ring. I explained how I was lost. He told me to get in the car…The man took me to his house. … He gave me food; he slept with me. I did not mind. This went on for 3 weeks. He gave me money, then to other men who paid me for sex. … these other men introduced where to get more clients. It was a big market. That has been my work.”

Most of the participants alluded to the practice of sex work in unpredictable forced flight. For instance, Meggie (she/her) fled from threats by community members in Meru and ended up in Nairobi’s downtown. She was stranded on the streets with street boys and later connected with male sex workers soliciting clients. Some were found at the ring road outside Hotel X and pimped by older sex workers. Like in Nairobi, in Mombasa, participants discussed how older MSM sex workers pimped them. Over and over the economic desperation and need shaped decisions about work and labor. Often in the stories, Nairobi and Mombasa were offered as destination points due to their bigger economies.

## Kinship, networks, and social systems

### (re)building nodes and networks

“The good thing about this [LGBTQ+] population is that there is connecting with each other. If you can locate one, you’ll find your way.”- *FGD gay individuals in Kisumu.*

Within such forced displacement, most queer people find themselves in the wild, which renders them socially vulnerable. Social support from social networks created in flight was essential in building a survival strategy and played a critical role in displacements and migration patterns. The networks emerged on the streets, and also newcomers in cities interacted with those already established in the networks, who were knowledgeable about survival strategies and clearly understood the importance of the collective in the pursuit of livability. Most of the study participants experienced these networks. They found each other and developed groups, among them the Freedom Corner group in Nairobi, and expanded to other groups. While networks tended to include LGBTQ+ people from different social classes, class and disparity issues represented a particular dynamic in networking. For instance, all Freedom Corner group members were sex workers. In Mombasa, groupings were much around class. Low-class met on streets. There was one meeting house in a middle-income estate, only second and first-class MSMs met here to socialize and discuss life issues. Some of them have invested in businesses. – *Fieldnotes, Mombasa, 2016-study 3*.

HIV made connecting much more accessible through networks. The flow of funding that accompanied the HIV response focusing on key populations resulting in a growth of local networks of organizations, including MSM-led organizations, to some extent, has been a catalyst for social connection and economic survival narrative for some queer people. These programs create link-up spaces where MSM and MSM sex workers link up, socialize, and participate in HIV interventions, including participating in psychosocial support. New arrivals are recruited into HIV interventions, and those who are HIV positive are assigned to peer educators, who give them HIV information, including treatment or prevention, and introduce them to survival tactics. In addition, peer educators are critical in helping new arrivals build nodes and networks, including creating HIV kinship. For instance, Jedida, a peer educator in Mombasa, explains how she created HIV kinship through peer education work. “They are my children I take care of. I give them HIV information, supply them with condoms and lubricants, and accompany them to health facilities. If they do not have money, I show them how to survive on sex work because it is an easy way to make money. I introduce them to clients, and they get money for food and pay for housing. I introduce them to HIV activities, where they meet other MSM. Usually, when they settle down, they visit me and bring me gifts.” – *IDI 2022- Study 5*. Many gay, bisexual, and transgender men link up in spaces they have created, or they patronized most.

### Respectability and reconnecting with kin

What emerges in this sub-theme is restoring connections and social credibility with family members. Despite being chased away and not being wanted, some LGBTQ+ people had a sense of restoring relation with their kin. They reflected on this as a sense of reconnecting, which came through buying respectability or death or simply others expressing desirability to reconnect. “They like our money, not us” was a statement summing up a discussion with MSM sex workers in Mombasa *(study 3)* on their relationship with family members. Abdi is one of those whose connection with kin depends on his money. At 10 am on a sunny Saturday, the first author came through Abdi’s house. While it was usual for the first author to visit the study participants, she was intrigued by Abdi’s expression of gratitude for the visit. Abdi response to the question why he continues to send money to his siblings despite the rejection:

“I’m very happy to see you. My family back home never calls to say hi. No greetings. But I support my family members financially. Even though I support them, none of them call to find out how I am doing or say hi. But they text a list of their economic needs for me to send money. I do send them the money, but they never say thank you. Once I called to ask if they had received the money, and the response was, did not you see the Mpesa (mobile money) message on your phone?’ That is what it is.” –*IDI Mombasa 2017 -study 3.*

Despite rejection by his family, he continued financial support to keep relationship with them. He explained, “I want to have a relationship with them, and if this is the only way to stay connected with them, then so be it. I work hard to keep this relationship by ensuring I have money to send to them when they send the lists.” - *Informal discussion Mombasa -study 3*. This strategy seemed to have worked. Abdi saddled with the care of the parents and siblings who had financial inability.

Like Abdi, those who were rejected were shown tolerance upon improvement of their financial means. Jamal explained how when life was tough, he tried to return home but was chased away by his mother. “I went back to my rural home, and when I knocked on the door, my mother chased me away … she told me to go away. I left and came back to Nairobi.” – *Fieldnotes study 1*. Jamal shared how improvement in his fortunes greatly changed his relationship with his mother and family members. He has been [accepted] by his mother and the rest of his family members, of course shouldering too heavy family financial obligations or responsibilities. Such were cases across study sites revealing familistic desires of LGBTQ+ individuals.

As mentioned above, some mothers are a source of support in the flight. However, some created an expectation for reciprocal support should the child secure financial stability. Kiddy (he/him/his) was one of the first queer people the first author met during her research work in Mombasa in 2015. Kiddy was often fondly spoke about her mother’s support. He stopped talking about the mother following her ‘unreasonable’ monetary demands on him. Like Abdi and Kiddy, economic diaries in study 3 revealed remittances to kin in rural areas topped MSM sex workers expenditures.

LGBTQ+ people who economically support community projects are celebrated and shown respect even in death, while those who are poor remain rejected. In 2023, the first author attended the funeral of Kark. Kark (from study1) was one of the few LGBTQ+ people who benefited from the local economy of NGOs. With a Bachelor of Arts degree, he worked as a senior staff member in a queer-led organization. Unlike three other queer funerals the first author attended, where it is the queer people who transport the body to the village for burial, Kark’s relatives and his pastor from his rural village came to collect his body from Nairobi. As the funeral procession moved from Nairobi to the rural area, the pastor praised Kark for his financial support to his church. It was clear from the eulogies that he invested heavily in supporting his family, including putting up a house for the mother, paying school fees for nephews and nieces, and providing financial support to his siblings and stepsiblings. He also supported the community’s church and paid school fees for children in his village. The community came out in large numbers for the burial. Eulogy speeches were packed with praise. The pastor conducted full burial rites for Kark. The queer community eulogized Kark and fundraised for a church project.

Meanwhile, in the other three funerals, tensions were evident between the queer community who took the body to the villages and the local community. All the dead were peer educators and sex workers who were economically struggling and had cut off links with kin. In such cases, it is the LGBTQ+ community who transports bodies to deceased original homes for burial.

Unlike Kark’s elaborate burial, Carol’s family did not come to take her body, but they offered a place for her burial. During Carol’s burial, tensions were observable between the queer community and the locals, including the relatives. Only a handful of villagers joined the queer community to bury Carol. Only the uncle, who chased her away, eulogized, expressing disappointment with Carol. He said, “He [Carol] is 29 years old; he has no family, no child, and no [heteronormative] wife. We have nothing to remember him by. He has left us with pain.” The priest conducting the burial lamented, “We do not know what he has been up to. He used to go to church at home, but we have not received any [church] letter from Nairobi.” *Fieldnotes 2016 study 1 case history participant.* As a periodic peer educator, Carol did not have money to support her family, as Kark did. “He had nothing,” noted one of the Freedom Corner group members. The LGBTQ+ organization sent money for food. Because of the tension, queer friends feared to eulogize. It is evident that financial ability is a key determinant in relations with kin. While those who had no money or were struggling economically cut off links, those with financial ability assuming financial responsibility bought respectability.

## Concluding discussion

Our findings showed that serial forced internal displacement of LGBTQ+ people from various social backgrounds and with different biographies in Kenya is precipitated by stigma and violence perpetrated across numerous levels of society. Understanding LGBTQ+ surveillance and displacement is vital to grasp how sociocultural norms and expectations are central to LGBTQ+ people’s displacement and suffering narratives in Kenya and maybe more globally. LGBTQ+ people become moving targets, constantly subjected to negative attitudes based on socially embedded norms and practices thus keeping them moving from one place to another for safety.

LGBTQ+ IDPs is shaped by various factors. In this study, push factors were located at the individual, interpersonal, community, institutional and structural levels. The push factors such as stigma, discrimination, violence, disclosure and concealment of identities resonate with other queer migration studies (Wilson et al., 2019; Lewis et al., 2023). (Bhagat, 2018; Granderson et al., 2019; Haase et al., 2023). At the individual level, LGBTQ+ people contend with disclosure corners and fears of rejection and oppression from others. There are reports of rejection, ostracization, isolation, and loss of social support and networks (Bhagat, 2018); in the interpersonal domain, there was a high level of victimization perpetrated by family members that are often rooted in institutional norms of patriarchy and misogyny. For example, the hegemonic patriarchy and misogyny are enforced by blaming mothers for “abnormal” children. Then, there is the violence enacted by evicting LGBTQ children and wives them from their home. These norms undermine LGBTQ+ people and their mother’s ability to integrate and be included into society; instead, they are forced into perpetual internal displacement. Community-level factors, such as community norms and surveillance (e.g., Swahili homes), further exacerbate displacement narratives among LGBTQ+ people in Kenya.

As this study shows, LGBTQ+ migration is marked by a myriad of struggles that LGBTQ+ people face in their daily lives. As was found in our study, internal mobility or displacement of queer persons is happening (Ritholtz, 2023; Bhagat, 2018), but remains a blind spot in research (Woensdregt, 2023; Ritholtz, 2023). Even though some LGBTQ+ persons migrate in search of friendships, and a sense of belonging (Stella et al., 2017; Gorman-Murray, 2009), interviews, and discussions in this study, revealed that pervasive negative attitudes combined with the criminalization of same-sex in Kenya, create conditions for forced migration of LGBTQ+ Kenyans. And that queer mobility urban pathway is the preferred displacement destination, with the potential for growth of queer spaces, social networks, and individual incomes (Ombagi, 2019; Woensdregt, 2023; Shio and Moyer, 2021). LGBTQ+ person’s displacement is permeated with challenges (Shio and Moyer, 2021; Ritholtz, 2023). Financial despair create numerous other challenges (Ritholtz, 2023; Shio and Moyer, 2021; Vogel, 2009). It emerged from this study that support is critical during forced displacement (Vogel, 2009; Shio and Moyer, 2021; Ritholtz, 2023). While humanitarian aid plays an acritical role in support of migrants, including LGBTQ+ migrants, in this study, humanitarian aid overlooks LGBTQ+ IDPs. As a result, the forced migration is imbued with safety risks, homelessness, health risks, disruption or lack of social networks and support, and lack of income and loss of social support (Held, 2023; Vogel, 2009; Piwowarczyk et al., 2017; Ritholtz, 2023). Where available, social support was critical assets leveraged during flights, including support from friends, organizations, and sometimes mothers (Ombagi, 2019; Woensdregt, 2023; Shio and Moyer, 2021). However, in circumstances where forced displacement intersects with moralization and criminalization of same-sex relations, it is impossible to galvanize social support from others, including family, community, and the state. These findings raise important considerations within the internally displaced persons (IDP) conceptual frameworks, highlighting crucial socioecological aspects of the LGBTQ+ IDP experiences (Bhagat, 2018; Bhagat, 2023; Quintero and Hari, 2022; Ritholtz, 2023; Gorman-Murray et al., 2014). This study also indicates that LGBTQ+ migration might be better served if examined through the lens of “forced displacement or migration,” given the non-voluntary aspects of flight and migration (Bhagat, 2018).

### Study limitations

We acknowledge the limitations of this research. The main restriction is that LGBTQ+ migration was not the main research question of the studies but rather emerged organically as studies unfolded. Thus, these experiences only “scratch the surface,” and additional studies are needed that explicitly examine LGBTQ+ migration and displacement. There is also selection bias in the sample population, given that it is skewed toward MSM, sex workers, and those who participate in LGBTQ networks, which may have different experiences compared to those who do not seek services and support. There is a need for greater diversity in sampling to include different socioeconomic backgrounds and representation across the LGBTQ+ spectrum, with a particular focus on lesbian and transgender persons. Even when measures were made to include all the LGBTQ+ spectrum in the political economy study (study four) and LGBTQ lived experiences study (study five), we still did not penetrate the middle- and upper-class LGBTQ+ population, who may be more stable.

## Conclusion

Inclusion of LGBTQ Kenyans in HIV interventions did not remove causes of forced migration. There can be little doubt that they face stigma and discrimination across levels of the socioecological model. In fact, in most cases, there has been multiplied grievances and anti- LGBTQ+ activities. Rethinking the internal displacement of queer people through the lens of coercion may better capture the lived experiences given the structural stigma and violence in which they live and cannot escape. Ethnographies may offer opportunities to deepen the understanding and fragility of LGBTQ+ internal displacement.

## Data Availability

The original contributions presented in the study are included in the article, further inquiries can be directed to the corresponding author/s.
